# A novel plausible mechanism of NSAIDs-induced apoptosis in cancer cells: the implication of proline oxidase and peroxisome proliferator-activated receptor

**DOI:** 10.1007/s43440-020-00140-z

**Published:** 2020-07-24

**Authors:** Adam Kazberuk, Ilona Zareba, Jerzy Palka, Arkadiusz Surazynski

**Affiliations:** Department of Medicinal Chemistry, Medical University of Bialystok, Mickiewicza 2D, 15-222 Białystok, Poland

**Keywords:** Cancer cells, Apoptosis, Non-steroidal anti-inflammatory drugs, Peroxisome proliferator-activated receptor, Proline dehydrogenase, Proline oxidase

## Abstract

Although pharmaco-epidemiological studies provided evidence for the anticancer potential of non-steroidal anti-inflammatory drugs (NSAIDs), the mechanism of their anti-cancer activity is not known. Several lines of evidence suggest that proline dehydrogenase/proline oxidase (PRODH/POX) may represent a target for NSAIDs-dependent anti-cancer activity. PRODH/POX catalyzes conversion of proline into Δ1-pyrroline-5-carboxylate releasing ATP or reactive oxygen species for autophagy/apoptosis. Since NSAIDs are ligands of peroxisome proliferator-activated receptor (PPARs) and PPARs are implicated in PRODH/POX-dependent apoptosis we provided a hypothesis on the mechanism of NSAIDs-induced apoptosis in cancer cells.

## Anticancer activity of NSAIDs

Non-steroidal anti-inflammatory drugs (NSAIDs) are a class of drugs commonly prescribed due to their wide spectrum of pharmacological effects. However they are preferred for the treatment of inflammatory diseases. The molecular mechanism of NSAIDs action is related to the inhibition of cyclooxygenases (COX-1 and COX-2), enzymes catalyzing the biosynthesis of prostaglandins (PGs) from arachidonic and linoleic acids. COX-1 is expressed constitutively in most mammalian cells and maintains homeostasis of some physiological processes, while COX-2 is induced in response to inflammation [1]. While inhibition of COX-1 evokes antiplatelet effect, inhibition of COX-2 has strong anti-inflammatory, antipyretic and analgesic effects [2, 3].

It is well established that inflammatory environment promotes cancer development. The mechanism of this process is due to increased levels of COX-2 and prostaglandin E2 (PGE_2_) [4–7] that promote proliferation, migration, invasion, and cell adhesion [8, 9]. According to these facts, medication with NSAIDs was associated with decreased risk of certain cancer types, particularly gastrointestinal tract cancers (gastric or colorectal cancer), lung, breast, and prostate cancers [10–14]. Clinical and pharmacoepidemiological studies provide evidence that aspirin and other cyclooxygenase-2 enzyme inhibitors lower recurrence of colorectal cancer by about 20% [12, 15, 16]. Another example is that regular, non-selective COX-2 NSAIDs treatment (i.e. aspirin and ibuprofen) caused a 69% reduction in the relative risk of lung cancer [17]. The explanation for the potential mechanism of anticancer activity of NSAIDs comes from studies on the inhibitory effect on cyclooxygenases that are frequently overexpressed in different types of cancer [18, 19]. Such a mechanism was observed in cultured HT-29 human colon cancer cells where apoptosis occurred after incubation with sulindac and sulindac sulfide, salicylate and other NSAIDs [20]. COX-2 inhibition attenuates also angiogenesis through expression of vascular endothelial growth factor (VEGF) and metalloproteinases [21]. However, some experiments show that the anti-neoplastic effect of NSAIDs is more complex and cannot be explained on the basis of cyclooxygenase inhibition pathway [22]. In human prostate cancer cell lines, PC3 and LNCaP which are lacking COX-2, the treatment with selective COX-2 inhibitor, celecoxib inhibited the growth of both cell lines independently of PGE_2_ level. The similar effect was observed in vivo [23, 24]. Other representative studies carried out using human colon cancer HT-29 cells expressing COX-1 and -2 and HCT-15 lacking both isoforms of cyclooxygenase confirmed prostaglandin-independent effects of NSAIDs. However, the concentrations of NSAIDs required for inhibition of COX and cancer cell proliferation are different [20, 25]. The concentration of NSAIDs required for inhibition of cell proliferation is much higher than those for inhibition of cyclooxygenases activity. Another evidence for COX-independent effect of NSAIDs was provided by studies on chiral centers of ibuprofen and flurbiprofen. When the drugs are *S*-enantiomers they evoke non-selective COX inhibition while *R*-enantiomers are deprived of both COX-1 or COX-2 inhibitory activity. However, both *S*- and *R*-enantiomers have the same anti-proliferative effects. It has been suggested that this effects of NSAIDs can be related to inhibition of cyclic guanosine monophosphate phosphodiesterases (cGMP PDEs) signaling, Wnt/β-catenin signaling, peroxisome proliferator-activated receptors, retinoid X receptors, IKKβ/NF-κB, PDK-1/AKT, Akt/mTOR signaling inhibition and AMP-activated protein kinase (AMPK) up-regulation [26–28].

Another possible pathway potentially involved in NSAIDs induced apoptosis in cancer cells is related to the activity of 15-lipoxygenase-1 (15-LOX-1). COX and LOX are the major enzymes responsible for polyunsaturated fatty acids metabolism. In vitro and in vivo studies indicated that gene expression of 15-LOX-1 and level of its main product, 13-hydroxyoctadecadienoic acid (13-S-HODE) is significantly decreased in adenomas or carcinomas comparing to normal mucosa [29, 30]. LOX is the main enzyme metabolizing colonic linoleic acid to eicosanoids. In-vitro experiments with colon cancer cells that have a different level of COXs expression show that NSAIDs (e.g. sulindac sulfone) can up-regulate 15-LOX-1 expression and increase the formation of 13-S-HODE—the main metabolic product of this enzyme. These effects were related to the apoptosis induction in colon cancer cells and LOX-dependent apoptosis was reversed by using caffeic acid—a 15-LOX-1 inhibitor. Interestingly when the cells were incubated with sulindac sulfone, caffeic acid and 13-S-HODE, apoptosis was significantly elevated but the substitution of 13-S-HODE by linoleic acid had no effect in this combination. One explanation of this effect can be a shift of substrate away from the COXs and toward the LOXs [31]. Another possibility could be the interaction between LOX activity and peroxisome proliferator-activated receptors (PPARs). Increased level of 13-S-HODE, in response to 15-LOX-1 activation can be responsible for significant down-regulation of peroxisome proliferator-activated receptor δ (PPARδ) in RKO and DLD-1 colon cancer cells. Linoleic acid as a substrate for 15-LOX-1 did not have the same effect alone. Further experiments proved that molecular mechanism for this effects is also related to 13-S-HODE direct binding with PPARδ and downregulation of its expression [32, 33] or even direct 15-LOX-1 to tumor suppressor protein (p53) interaction independently of lipoxygenase enzymatic activity [34]. Another important fact is that products of 15-LOX-1 are well-known ligands of peroxisome proliferator-activated receptor γ (PPARγ). In vitro model with macrophages or HCT-116 and KO1 cells proved that products of lipid oxidation, particularly 13-S-HODE are effective PPARγ activators and can promote apoptosis. Some studies have revealed that native LDL had no influence on reporter PPARγ activity even when high concentrations were used. When PPARγ reporter activity was stimulated at low concentration of 13-S-HODE [35] it contributed to 70% PPARδ downregulation [32]. Therefore, it has been suggested that NSAIDs-dependent pro-apoptotic activity is mediated by PPARs. Although native LDL has no influence on PPARγ activation, such an activity evokes oxidized LDL (oxLDL) that mediates PPARγ transcription [35].

All those signaling pathways are linked to PPARγ-dependent functions. NSAIDs-dependent activation of PPARγ down-regulate pro-survival pathways (e.g. Wnt/β-catenin and Akt/mTOR signaling) and up-regulate pro-apoptotic signaling (e.g. AMPK or LOX-1 activated PPARγ and PRODH/POX).

## Molecular polymorphism and function of PPAR

The peroxisome proliferator-activated receptors (PPAR) are ligand-dependent transcription factors belonging to the nuclear hormone receptor family. PPARs can regulate the transcription of multiple genes in response to activation by natural or synthetic ligands. Although PPARs regulate lipid metabolism, glucose homeostasis, and adipogenesis [36], they can also affect inflammation, proliferation, differentiation, and carcinogenesis [37, 38]. Activation of PPAR requires heterodimerization with retinoid X receptors (RXR) to form PPAR/RXR complex which binds to specific DNA fragment called PPAR response element (PPRE) in a target gene [39]. Three different isoforms of PPAR are known: PPARα (NR1C1), PPARβ/δ (NR1C2) and PPARγ (NR1C3) and they all are encoded by different genes [40–42]. Isoforms α, β/δ, γ are differentially expressed in embryonic and adult tissues [43]. PPARα, PPARβ/δ and PPARγ have similar structural and functional domains specific for this group of the nuclear receptors. Four of them: A/B, C, D, and E/F domains have been determined [44]. N-terminal A/B ligand-independent transactivation function domain (activation function—AF-1) and its activity is related to phosphorylation or sumoylation. Depending on receptor isotype it can be active or non-active in the basal state. DNA-binding domain C contains two highly conserved zinc finger motifs which bind hormone response element (HRE) and recognizes the promoter region of target genes known as PPRE. Flexible hinge region with activity of docking site for cofactors is in D domain, and E/F domain with ligand binding domain (LBD) which is C-terminal plays important role in activation of PPAR and PPRE interaction. This results in amplifying of target genes expression with important role of activation function-2 (AF-2) [45, 46]. If the ligand is not present, PPARs can heterodimerize with RXR and bind to promoter region of target gene and recruit corepressors like N-CoR or SMRT resulting in inhibition of gene expression. Conformational changes in PPAR structures (due to ligand binding), lead to corepressor dissociation and interaction with coactivator initiating gene expression [47].

PPARα was the first cloned isoform of this receptor family [48]. It regulates the genes expression involved in cholesterol transport and free fatty acids (FAs) metabolism through the β-oxidation and peroxisomal pathways [49, 50]. PPARα serves as the main regulator of lipid metabolism in the liver [51]. It is highly expressed in tissues catabolizing fatty acids such as skeletal muscle, brown adipose, kidney, heart, and liver [52, 53]. Moreover these receptors are expressed in vascular and immune cells [54] and also in the hippocampus and hippocampal neurons [55]. Activation of PPARα by statins like simvastatin leads to increased neurotrophins expression which is important in processes of learning and memory [56].

PPARβ/δ have ubiquitous localization with high expression in the intestine, liver, abdominal adipose tissue, skeletal muscle, liver. They regulate glucose and cholesterol level in blood and are involved in lipid metabolism [57, 58]. PPARβ/δ function as a transcriptional repressor in its unliganded state what differs them from PPARα and PPARγ. Repression of basal transcription as well as PPARα- and PPARγ-mediated transcription can occur due to unliganded PPARβ/δ through the corepressor recruitment. PPARβ/δ can inhibit PPARα and PPARγ activity by isotype-specific repression due to PPRE sites competition [59]. Activation of PPARβ/δ has also pro-tumorigenic effect in breast cancers. Fatty acid-binding protein 5 (FABP5) interaction with PPARβ/δ mediates epidermal growth factor receptor (EGFR) dependent cell proliferation. GW501516, GW0742, and L-165041 are the synthetic ligands with very high affinity to β/δ isoform at low concentrations (1.1 nM for GW501516, 1.0 nM for GW0742 and 50 nM for L165041) and significant selectivity over the other isoforms of PPARs [60].

Selective PPARβ/δ agonist GW501516 accelerated tumor formation in mice while inverse agonist inhibited PPARβ/δ targeting genes related to MDA-MB-231 cell invasion. High expression of PPARβ/δ in MCF-7 enhanced cell migration and increased resistance to endoplasmic reticulum stress conditions as low glucose and hypoxia. This suggests their important role in the adaptation of breast cancer cells to different micro-environmental stress conditions [61]. It was found that overexpression of PPARβ/δ in human cancers promotes tumor growth by increasing VEGF expression and activating PI3K-Akt signaling supporting cell survival [62]. Moreover, higher expression of PPARδ in chronic lymphocytic leukemia (CLL) and other hematologic cancers was found to support growth in stress conditions as hypoxia, low glucose, and exposure to cytotoxic drugs. Synthetic PPARδ antagonists and genetic deletion of PPARδ reversed its growth supporting activity [63].

PPARγ is widely expressed in brown and white adipose tissue, spleen and large intestine [64, 65]. PPARγ has three isoforms which are transcribed on the same gene but undergo control of different promoters [66]. γ1 and γ3 have different mRNA but the protein is the same for both isoforms [64]. γ1 is present in brown and white adipose tissue, large intestine, immune cells, pancreas, liver, small intestine and kidney [67]. Low level of expression is in central nervous systems like in astrocytes, neurons, microglia, and oligodendrocytes. Isoform γ2 is present only in adipose tissue and differs from others due to an additional 30 amino acids on the N-terminal site [68, 69]. Targeting PPARγ was used for the type 2 diabetes treatment with thiazolidinedione (TZD) class of drugs i.e. rosiglitazone, troglitazone, and pioglitazone [70]. PPARγ agonists have been also shown to function as an anticancer factors, especially for obesity related cancers as a prostate, breast, colon, liver, thyroid, lung, and pituitary cancers [71]. It has been linked to the anti-inflammatory activity of PPARs. Anti-inflammatory effects of PPARγ were observed due to inhibition of tumor necrosis factor α (TNF-α), interleukin 1β (IL-1β), interleukin 6 (IL-6) and PGE2 production [72]. PPARγ is expressed in breast adenocarcinoma, human liposarcoma and some of colonic cancer cell lines [73]. Activation of PPARγ contributes to lowering the level of angiogenic factors and reduction in migration and proliferation of endothelial cells [74]. Ligand activation of PPARγ by troglitazone promotes TRAIL-induced apoptosis in human lung cancer via autophagy [75]. Adenovirus gene transferred SNU-668 gastric cancer cells with overexpression of PPARγ presented significant the growth inhibition and apoptosis activation due to strong IGFBP-3 upregulation. Insulin-like growth factor-binding protein-3 IGFBP-3 is a tumor suppressor gene, independent of IGF signaling [76]. Some reports proved that activation of PPARγ can inhibit the growth of ovarian cancer by suppressing proto-oncogene B-cell lymphoma 3-encoded protein (BCL3) in response to microRNA-125b (miR-125b) tumor suppressor upregulation. In fact in bladder, breast, ovarian and lung cancer, it was observed that miR-125b tumor suppressor level was downregulated and this affected IGF, PI3K/Akt/mTOR, and mitogen activated protein kinase (MAPK) signaling pathways [77]. Although the PPARγ activation contributes to the pro-apoptotic phenotype of cancer cells, the molecular mechanism of this process in still unknown. One of the enzymes regulated by PPARγ and involved in cell death is proline oxidase (POX).

## PRODH/POX-dependent apoptosis

Proline oxidase (POX) also known as a proline dehydrogenase (PRODH) is an inner mitochondrial membrane flavin-dependent enzyme which catalyzes the conversion of l-proline to ∆1-pyrroline-5-carboxylate (P5C). This process donates electrons through flavin adenine dinucleotide (FAD) to the electron transport chain for ATP generation. From this point of view the activity of PRODH/POX promotes cell survival. However, in certain conditions the same electrons are directly transferred to oxygen forming superoxide radicals and other reactive oxygen species (ROS) leading to programmed cell death—apoptosis. However depending on environmental conditions superoxide radicals generated by PRODH/POX may promote pro-survival autophagy [78, 79]. In healthy human tissues PRODH/POX activity may vary depending on the type of tissue. High expression is present in liver and kidney while in brain and heart is low. In most of the other tissues PRODH/POX is undetectable [80, 81]. Variety of cancer cell lines under low oxygen level (hypoxia) have increased PRODH/POX activity, compared to normoxia [82]. In stress conditions like glucose depletion and hypoxia it was observed PRODH/POX upregulation through AMPK activation promoting cancer cell survival. In low glucose conditions with or without hypoxia PRODH/POX was found to induce adenosine triphosphate (ATP) production, however appropriate glucose level and hypoxia resulted in protective autophagy with PRODH/POX-mediated reactive oxygen species (ROS) generation. These findings suggest the important role of tumor microenvironment in PRODH/POX-dependent functions [83].

Proline oxidase expression and function can be regulated by different factors. One of this factor is PPARγ. Stimulation of PPARγ by its ligand as troglitazone increased the binding of PPARγ to the PRODH/POX promoter and triggered its expression. Furthermore troglitazone treated cancer cells presented significantly increased PRODH/POX mRNA level (in dose depended manner) in comparison to non-treated cells. These data suggested the important role of PPARγ in PRODH/POX expression, intracellular ROS generation, and cell death. A selective inhibitor of PPARγ GW9662 in combination with drugs mentioned above recovered this effect but had no effect alone [84, 85]. In the model of colon cancer cells with doxycycline regulated PRODH/POX expression activation of PRODH/POX significantly reduced COX-2 expression, PGE_2_ level and induced ROS generation leading to strong proapoptotic effect. This phenomenon was reversed by the treatment with PGE_2_ and also manganese superoxide dismutase (MnSOD), a mitochondrial enzyme, which neutralizes superoxides. Moreover, PRODH/POX-dependent down-regulation of COX-2 was partially reversed by EGF through Wnt/β-catenin and EGFR signaling mechanism [86].

In DLD-1 colon cancer cells expressing PRODH/POX, supplementation of culture media with 0.5 mM of l-proline resulted in mitochondria-mediated apoptosis due to, caspase 9 activation, cytochrome c release and nuclear condensation/fragmentation independently of p53 contribution [80]. Studies of recent years have established an important role of proline in cancer cell metabolism. Understanding the role of this amino acid in regulation on cell survival and death focused therefore on enzymes involved in proline cycling. PRODH/POX and pyrroline-5-carboxylate reductase (P5CR, a.k.a. PYCR) are of special interest. Ornithine or glutamate are substrates for proline synthesis and both of them leads to l-glutamate-γ-semialdehyde (GSAL) production, which can be converted reversibly and spontaneously into P5C. Transformation of ornithine to GSAL is possible due to ornithine δ-aminoacid transferase (OAT), while P5C synthase (P5CS) catalyzes the process of P5C synthesis from l-glutamate with GSAL intermediate. These steps of proline biosynthesis occur in mitochondria. If P5C is transferred to the cytosol, then it can be reduced to proline. This process is catalyzed by P5C reductase (PYCRL) which is the NADPH-dependent enzyme. A similar reaction can take place in mitochondria, but in this case different isoform of this enzyme as PYCR1 or PYCR2 are involved. It was proved that knockdown of PYCR1 can be also responsible for reduced cell proliferation in liver cancer [87]. Proline metabolite—glutamate by further conversion to αKG by glutamate dehydrogenase can enter the TCA cycle and contribute to cellular energy production. Another issue is the role of proline cycle in cell proliferation and biomass production through the link to the pentose phosphate pathway. During this step some precursors of nucleotides required for DNA and RNA synthesis are produced [88, 89]. However proline for PRODH/POX-dependent apoptosis is also derived from collagen degradation products. Proline and hydroxyproline constitute about 25% of residues in collagen [90]. The most important process supporting intracellular proline is regulated by prolidase.

## The role of prolidase in PRODH/POX-dependent apoptosis

Prolidase (PEPD, peptidase D or iminopeptidase) is imido-dipeptidase or imido-tripeptidase localized in the cytoplasm [91, 92] and its function is to cleave imido-peptides with C-terminal proline or hydroxyproline [93]. They are derived mainly from collagen degradation products [94, 95]. In the α1 subunit of type I procollagen, proline forms 119 bonds with glycine and in α2 subunit such a doublet occurs 106 times. Although in matured collagen proline is mostly hydroxylated. Un-hydroxylated proline in glycine–proline (gly–pro) doublet occurs 25 times [95]. Therefore collagen degradation significantly contributes to intracellular proline concentration. It is known that prolidase activity is an important factor for proline recycling for collagen re-synthesis and therefore the enzyme plays a step limiting role in the regulation of collagen biosynthesis. The importance of this iminopeptidase in regulation of collagen biosynthesis was documented in fibroblast treated with proline metabolite—P5C [96], anti-inflammatory drugs [97], during experimental fibroblasts aging [98], experimental chondrocytes inflammation [99], activation of integrin receptor for type I collagen [100], in fibroblast-derived from osteogenesis imperfecta affected patients [101] and in several cancer tissues [102–104]. It was also found that prolidase may act at the level of transcription factors regulation. In colorectal cancer cells prolidase overexpression was correlated with increased levels of nuclear hypoxia inducible factor 1α (HIF-1α) and HIF-1α-dependent gene products like a vascular endothelial growth factor (VEGF) and glucose transporter-1 (Glut-1)—important factors in cancer progression [105]. Suppressed proteasomal degradation of HIF-1α and increased HIF-1α transcriptional activity occurs when HIF prolyl hydroxylase activity is inhibited by proline. The increased HIF-1α transcriptional activity is due to increased concentration of cytoplasmic proline, as a result of prolidase overexpression. Activation of HIF-1α related pro-survival signaling pathways undergoes through inflammatory and pro-angiogenic genes (eg. COX-2, TNFα, IL-1, NFκB, VEGF) [106]. It suggests that prolidase activity plays important role in regulation of HIF-1α-dependent functions.

All these data suggest COX-independent mechanisms of NSAID-dependent apoptosis in cancer cells. Until now numerous investigations were conducted to confirm that cancer cells treatment with NSAIDs are associated with downregulation of oncogenic factors expression and up-regulation of apoptosis pathway with significant role of the PPARγ [12–16]. Since NSAIDs are ligands of PPARγ and PPARγ induces PRODH/POX-dependent apoptosis, this sequence of events may represent the mechanism of anticancer activity of NSAIDs.

## Conclusions

**Fig. 1:**
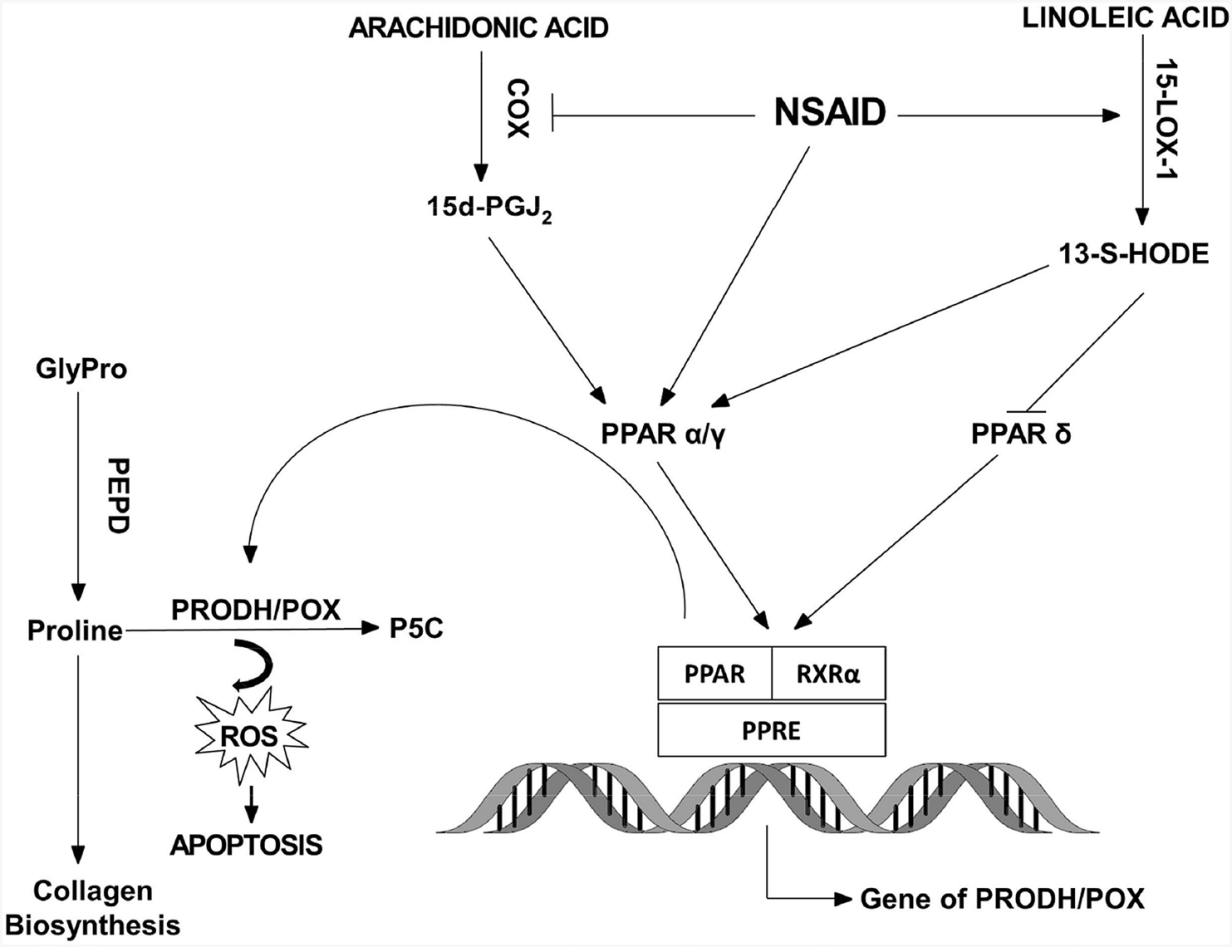
The potential mechanism of NSAID-dependent apoptosis in cancer cells

Studies of last decade provided evidence for the role of PRODH/POX and PPARs in the regulation of apoptosis/autophagy in cancer cells. PRODH/POX expression is often down-regulated in various tumors, limiting mitochondrial proline degradation and PRODH/POX-dependent apoptosis. NSAIDs were shown to stimulate the transcriptional activity of PPARα/γ that are well-characterized PRODH/POX inducers. However, the critical factor for the PRODH/POX-induced apoptosis is proline availability that depends on the activity of prolidase (enzyme supporting cytoplasmic proline level) and the intensity of collagen biosynthesis (proline utilizing process). Although specific environmental conditions may affect PPARs and PRODH/POX it seems that NSAIDs activate PRODH/POX-dependent apoptosis through PPARα/γ. The hypothesis is outlined in Fig. 1.

NSAIDs up-regulate PPARα/γ directly or indirectly (by 15-LOX-1-dependent generation of 13-S-HODE). Up-regulated PPARγ induces transcription of PRODH/POX and subsequently conversion of proline into P5C, generating ROS-inducing apoptosis. The process requires proline availability that is dependent on the activity of prolidase (proline supporting enzyme) and collagen biosynthesis (proline utilizing process). The role of other outlined NSAIDs-dependent pathways in the PRODH/POX-dependent apoptosis are unknown.
